# “Communitas in Crisis”: An Autoethnography of Psychosis Under Lockdown

**DOI:** 10.1177/10497323211025247

**Published:** 2021-06-26

**Authors:** Alison Fixsen

**Affiliations:** 1 University of Westminster, London, United Kingdom

**Keywords:** autoethnography, psychosis, lockdown, psychiatric unit, peer relationships, qualitative, autoethnography, UK

## Abstract

In this article, I use autoethnography to examine time spent on an acute psychiatric ward during the COVID-19 lockdown. I employ the device of “communitas in crisis” to emphasize the precarious nature of this experience and the extent to which, for myself at least, informal social interactions with fellow patients and “communitas” were significant features of my hospital experience and subsequent discharge. I suggest that a lack of emphasis on inpatient to inpatient relationships in the recovery literature is an omission and a reflection of psychiatry’s authority struggles with both service users and professionals, along with a general perception of psychosis as individual rather than as a socially constructed phenomenon. I also suggest that, especially in the wake of greater social distancing, mental health and social services should safeguard against psychological and social isolation by creating more spaces for struggling people to interact without fear or prejudice.

## Prelude

*“Morning, MORNING!!” The monologue continues as Sue, a petite middle-aged lady in her 60’s with learning difficulties, shouts out full blast as she marches down the corridor outside my bedroom. No doubt Sue has her ancient radio pressed to her ear as she is very hard of hearing- hence there is also the added joy of an untuned set blasting out some kind of breakfast news program. Having slept on my left-hand side all night to avoid the torch flashes waking me every half hour through the night (when the ward duty nurse checks the patients’ breathing) my leg has gone numb, still I stretch an arm out to check the time on my iPhone. Once more I awake sad to be here, but somewhat grateful for the companionship and help that has been offered to me. I’m in a psychiatric ward somewhere in England. It’s a very strange place to be especially during COVID-19 lockdown. On day one I was so out of it I couldn’t work out what was going on. Were the nurses real or just phantoms aimed at punishing or infecting me? I believed myself transported to a modern Bedlam, intensified by a pre-Christmas excitement which expressed itself in tinseled hairdos, Christmas carols and patients and nurses reveling in the prospect of food parcels and presents from loved ones. In my early state of misery it seemed ironic that the nurses were constantly appearing in my bedroom doorway to introduce themselves, only to be sent away or chastised for their over exuberance or not wearing their masks properly. A week or so in and I’m getting accustomed to most things except the noise level. The ridiculously high corridor ceilings create a cathedral like echo, such that every conversation becomes a chorus of undecipherable words. In my original paranoid state I was sure that I was not only imagining this whole scenario but going deaf to boot.*

## Introduction

In this article, I use autoethnography to interpret my experiences as a patient in a psychiatric ward during the COVID-19 lockdown and to explore the nature of informal relationships between patients on such wards. Interpretive autoethnography begins with the life experiences of the writer to move out to culture, discourse, and ideology (Denzin, 2014) and also to address injustices and to speak out for the voiceless (Zapata & Sepúlveda, 2017). Two key questions guided this study. The first, a personal one, concerns my journey in and out of psychiatric care; how best to comprehend and interpret it? To borrow Taylor’s (2014a) words, waking up in a mental asylum is not something you plan for. More accustomed to being on the other side of the health equation, finding myself incarcerated in an acute mental health unit came as a shock. I cannot say how many times I heard the phrase “it can happen to anyone,” and while I denied this fact at the time, I increasingly believe it to be true. The second question concerns the peer-relational elements within mental health discourses and discussions; why have they been side-lined in the recovery literature and what insights can be gleaned from traditional ethnography concerning the significance of such relationships in institutional settings?

The article is divided into the following parts. First, I discuss the rise in mental health problems in the context of this study, which was the COVID-19 pandemic. I then discuss patient experiences of treatment for psychosis and different conceptualizations of recovery. In particular, I point to the under-documentation of inpatient to inpatient relationships in the literature. Then, I explain my conceptual framework and methodology. Thereafter comes my narrative of time spent on a psychiatric ward, and finally, I discuss what I regard as major issues arising from this study. Although for conventional purposes I use terms such as mental health “disorders” and “psychosis,” my intention in this article is to emphasize the problematic nature of psychiatric labeling. I draw on a tradition of critical literature that challenges “myths” around mental illness (Cohen, 2015, 2016; Szasz, 1974) and conventional constructs of psychiatric settings to seek out different interpretations of what it is like to be treated under a mental health system. In writing about my own and others’ experiences of psychiatric care, I use words such as “mad” and “crazy” at times in dark humor but largely to challenge the stigma attached to such terms.

### Mental Health and the Pandemic

According to the Health Foundation, mental health disorders account for around one-quarter of the burden of health conditions in the United Kingdom (Marshall et al., 2021). Added to this, the recent COVID-19 pandemic is believed to have caused a parallel epidemic of fear, anxiety, and depression (Vindegaard & Benros, 2020; Yao et al., 2020). In particular, several authors have noted or reported the risks posed by the pandemic of reactive psychoses triggered by pandemic stress (Valdés-Florido et al., 2020) in people with a previous history of, or susceptibility to, psychosis (D’Agostino et al., 2020; Kozloff et al., 2020) and the burden this places on patients and the medical profession (Fiorillo & Gorwood, 2020). Features associated with psychotic disorders include, but are not limited to, delusions, hallucinations, disorganized behaviors, and cognitive impairment, all of which can result in higher incidences of homelessness, congregate housing, and other factors that place individuals at higher risk of infection (Kozloff et al., 2020). In one systematic review, a variety of factors were associated with higher risk of psychiatric symptoms and/or low psychological well-being, including female gender, poor self-related health, and relatives with COVID-19 (Vindegaard & Benros, 2020). The pandemic may also have exerted a profound effect on the treatment provided by acute mental health services. According to one study, in comparison to 2019, the number of home crisis resolution and inpatient referrals declined from March to April 2020, while hospitalized patients were significantly more often compulsorily detained under the Mental Health Act. Fifty-two percent of these patients had been diagnosed with a nonaffective psychotic disorder (Abbas et al., 2021). Although the reasons for this may be obscure, it is indicative of more extreme responses to pressures on both public and crisis agencies at this time.

### Patient Experience of Treatment for Psychosis

Introduced throughout England and Wales in 1983, the Mental Health Act sets out when people can be detained and hospitalized for mental health treatment against their wishes. While some studies on patient experience of hospitalization for mental health issues highlight inpatient services as facilitative to recovery (Summers & Happell, 2003), being detained under the Act can be a highly traumatic event. As suggested in the literature and this autoethnographic account, psychiatric facilities are busy and noisy, and the forced detention or management of distressed or angry patients can be disruptive and alarming for patients. In 2017, an independent review of the Mental Health Act was announced with the intention of addressing concerns about rising detentions, including the disproportionate number of people from Black and minority ethnic groups detained under the Act (National Survivor User Network [NSUN], 2021). However, criticism of mainstream psychiatry and its paradigms and methods goes back much further.

Alongside a growing body of critical treaties on stigma, labeling, and treatment of mental illness (e.g., Foucault, 1961/2001; Goffman, 1963; Szasz, 1974), a psychiatric survivors’ movement arose, inspired by the civil rights movements of the 1960s and 1970s and by stories of ex-patients of alleged psychiatric abuse (Oaks, 2006). Since then, the movement has fragmented and diversified, although common themes among “survivors” are “talking back” to the power and expectations of psychiatry (Morrison, 2009, p. x), resisting labels, rights protection, and self-determination. Local groups and national organizations continue to provide mutual support and to promote the rights of current and former mental health service users to have a voice (Davidson & Roe, 2007; Morrison, 2009). While longitudinal clinical research of serious mental breakdown has shown that improvement is as common, or more so, than progressive deterioration, the survivor movement has suggested a second meaning of recovery, which directly refers to a person’s rights to self-determination and inclusion in community life *despite* suffering from degrees of mental illness (Davidson et al., 2005). In other words, recovery no longer needs to constitute “normality” (my own term here) but can represent different ways of being, which cause minimal suffering and maximum agency for the individual and those who have relationships with them.

The abovementioned approach, while patient-driven, still largely supports an individual or personal model of recovery from mental ill-health (Price-Robertson et al., 2017), potentially obscuring the interpersonal contexts that are an essential aspect of all recovery and empowerment narratives. Another body of literature focuses on the role of peer support in facilitating recovery. Most of this concerns the structured or orchestrated variety, whereby peer support volunteers or workers (PSWs) employed in mental health services assist people in the community as a means of reducing hospital admissions (Pfeiffer et al., 2011) or support self-efficacy in people living with long-term conditions (McLean et al., 2012).

There is also a body of literature that documents people’s own experiences of being diagnosed and treated for psychosis, including first-episode psychosis. Examples of this are Davidson and Johnson’s first-person account of a dialogue between Johnson (herself someone who experiences psychosis) and Davidson (a practitioner endeavoring to better comprehend and support people who experience psychosis). Their dialogue focuses on the concept of ontological security that is required to address a basic loss of personhood which appears to be a significant source of the distress and disability associated with psychosis (Davidson & Johnson, 2014). In contrast to the mainstream psychiatric disregard of subjectivity, service users’ accounts focus on the understanding of psychosis both as an aspect of self and as precipitated by societal pressures. Campbell (1996), in his account of psychiatric admission and distress, describes his first crisis as not a medical but a “moral event, a moral failure . . . none of the important implications have been medical ones.” (p. 183). Johnston (2020), whose autoethnography traces his struggles with psychosis, arrest, psychiatric institutionalization and recovery, makes it clear that the stigma of mental illness does not necessarily diminish over time; indeed, the shocking realization that such a thing could have happened to one leaves a lasting and possibly indelible psychological and social mark.

In her autobiography of years spent in the asylum system, Taylor (2014b) suggests that mainstream psychiatric thinking retains its focus on correcting neurochemical imbalances, ignoring the need to explore complexity and depth and other tools required to navigate everyday life. “Today I am no crazier than I need to be to negotiate modern life,” she asserts (p. xii). One implication is that, if “madness” is a result of disconnection between feelings, thoughts, and the external world, then psychiatric units and their inhabitants may provide the context that can be required to reconnect. In my own case, the beginning of COVID-19 and my isolation from normal social and work routine provided the context of (but not necessarily the reasons for) my physical and mental breakdown. As in Gray (2019) personal account, I regard the friendships formed in hospital to have been highly significant in aiding my recovery during an acute episode of psychosis. In Gray’s words:

Befriending other people, getting their support, praying for others, and engaging in group activities, all aided in my recovery. By taking part in group activities like creative writing or music therapy, I became closer to *people* (not as a diagnosis of schizophrenia, bipolar disorder, or depression but as human beings with complex problems, emotions, and difficult past histories). (Gray, 2019, p. 283)

Why the social dimension of recovery, and in particular the role of inpatient to inpatient relationships, remains under-documented may have something to do with the nature of psychiatry and its authority struggles. Factors such as the expansion of the *DSM* (and of subsequent psychiatric labeling) and the general medicalization of modern life have resulted in an increasing “psychiatrisation of personal and public life” within the Western society (Cohen, 2016, p. 1) and all that implies. As the political and economic power of psychiatry has expanded, so its hegemony has been more contested, resulting in uncertainty outside and within the profession (Cohen, 2015, 2016) In one small-scale study by Katschnig, the majority of psychiatrists regarded the profession as having a negative public image, while three-quarters were concerned about their status in the medical profession. In addition, most were concerned about the validity of psychiatric diagnoses and current treatment strategies themselves (Katschnig, 2010).

From a constructionist perspective, a diagnosis represents a “focal point at which numerous interests, anxieties, values, knowledges, practices and other factors merge and converge” (Jutel & Nettleton, 2011, p. 794), a moral indictment ensuring that “the individual now inhabits an illness” (Klinkenborg, 1994). In practical terms, definitive clinical diagnosis remains the province of the psychiatrist as “medical expert,” with pharmaceutical solutions omnipresent for serious mental illness (Cohen, 2015, pp. 3–4). This drug-focused approach to the psychiatric consultation may feel at odds with the patient’s experience and wishes. In one review of first-episode psychosis, participants described a range of unhelpful responses from health professionals, a primary one being psychiatrists’ focus on narrow discussions around medication, with little time devoted to other concerns including side effects of these drugs (Griffiths et al., 2019). Personal experience as an inpatient further suggests that, without the say-so of the psychiatrist, nurses and pharmacists will rarely alter a patient’s treatment regime—although when it comes to administering drugs to reluctant patients, medics’ techniques of persuasion can vary considerably. This is far from suggesting that other forms of treatment do not exist on psychiatric wards. Even so, most studies have focused on structured and one-to-one therapy (such as occupational therapy and psychotherapy; Lloyd et al., 2008) rather than the role played by inpatient relationships in survival/recovery trajectories. Psychiatric wards are social living spaces, and for a social scientist such a myself, the institutional rituals within them and their potential for change were of interest to me.

### Conceptual Framework

With its emphasis on group ritual and symbolism, ethnography, and its offspring autoethnography, provides alternative means to interpret the experiences of those in institutional settings to that of standard clinical literature. For my conceptual framework, I have borrowed from Victor Turner’s concept of “communitas,” by which I mean the aspect of shared rituals which allows for a kind of shared experience that is significant for being both temporary and relatively status-less, (examples being amusement, disapproval, or boredom). This deeply shared and interpersonal experience is central to traditional ethnography, yet it is frequently overlooked in individualistic Western cultural settings where values of independence and personal achievement are emphasized (Price-Robertson et al., 2017). As a phenomenon, communitas is most likely to occur when a group of persons’ previous status has been temporarily or permanently eroded (V. Turner, 1974). “Betwixt and between” (V. Turner, 1967) points of entry and exit into their separate worlds, members of this temporary community share, for a brief time, some common meaning or experience. Communitas, as Turner conceived it, forms part of a rite of passage, the latter consisting of three distinct but interlinked phases: separation (i.e., detachment of an individual from an earlier social structure), liminality (where limen signifies a threshold), and aggregation (when the passage is completed; V. Turner, 1969). It is during the liminal or transitional phase, “betwixt and between” two cultural states, that communitas can arise. This “in-between” place is a “symbolic domain that has few or none of the attributes of [the person’s] past or coming state” (V. Turner, 1974, p. 232)

As I hope to illustrate in the following account, the acute psychiatric ward represented for me a “communitas in crisis,” a transitory place in which I and my fellow inmates, found themselves removed from their households, stripped of their customary freedoms, relatively status-less and cohabitants in a social world in which others (the nurses, support workers, psychologists, etc.) attempted to “re-aggregate” their inner and outer worlds.

### Method

As a methodology, autoethnography employs personal experience, insider knowledge, and existing research to understand and critique cultural experience, break silence, and navigate through confusion, pain, uncertainty. and anger (Ellis & Adams, 2014). Both process and creative product, I chose interpretive autoethnography as a means of personal expression and social critique which, by articulating experiences of phenomena generally assigned clinical diagnostic terms, can highlight issues that are commonly dismissed as being too subjective to be of “scientific” interest and to address injustices and problems that affect those lacking a voice (Fixsen, 2016; Zapata & Sepúlveda, 2017). When it comes from the heart, Ellis (1999) asserts, autoethnography inevitably includes the researcher’s vulnerable self, producing evocative stories that “create the effect of reality” (p. 996). In illness autoethnographies, the narrative is especially intimate, with the revelation of mental illness leaving the author particularly vulnerable to self and other critique (Richards, 2008). It takes courage—and maybe a touch of foolhardiness—to write an honest account of your weakest and darkest moments and to cast it to one’s potentials critics (Smith, 1999). Yet, there are few recognized platforms of articulation for those who have been through serious mental health distress. For myself, the experience of hospitalization had been sufficiently vivid, and my interactions with fellow patients were so memorable that I wanted others to appreciate these experiences.

The circumstances leading me to this study were not anticipated. My journey into care was swift, and besides the portraits of patients I rapidly sketched to pass my time in the hospital, my “data collection method” rested on what Ellis (1999) has termed “emotional recall,” whereby the researcher imagines being “back in the scene emotionally and physically” (p. 675). As a heuristic tool, emotional recall can be used as a key to capture the complexity of emotions both in the present and as remembered (Gariglio, 2018). Fox’s (2010) study of what is it like to have a parent die of Alzheimer’s disease illustrates how one’s emotional recall of pleasant and painful memories and experiences can be translated and shared autoethnographically and poignantly. Defenbaugh (2008) used this method to explore her own experiences of naming and renaming illness in the medical community and how it felt to be voicelessly shunted along a system in which medical practitioners remain “the voice of medicine” (p. 1403).

### Ethical Considerations

This is an interpretive autoethnography, and accounts of people and events in this article are told with the aid of my emotional recall alone. Events were recorded very soon after leaving the hospital and are entirely anonymous, with the use of pseudonyms throughout. Due to the unforeseen circumstances of the events, informed consent was not applicable; however, participant and staff permission was orally granted for in situ sketches of participants (used as emotional recall devices only). To check for general consistency and readability, I sent the first draft of my paper to two close family members for their comments.

Beyond what is contained in this article, no documentation was used or stored concerning participants, staff, or institution. Other actions taken to ensure that participants’ identities were not disclosed was to paraphrase any conversations or remarks made by those mentioned in the study; omit any identifying details concerning people’s appearance, age, and place of residence; and give only very brief descriptions of persons and surroundings. Events of an especially graphic or disturbing nature have been omitted from the narrative.

According to the criteria set out by the U.K. Research Medical Council, this study does not require ethical approval from an NHS Research Ethics Committee as it is not a clinical trial; no research was conducted on participants in disclosed locations; it did not involve recruitment of NHS staff as participants; being entirely observational, it did not require the collection of information from participants; and it does not involve the processing of confidential information on patients outside of the care team without consent (Medical Research Council, NHS Health Research Authority, 2021).

## Narrative Account

It is six-thirty on a cold, dark winter’s morning. An hour and a half to wait until breakfast. Two hours until my next lot of medication. I have come to accept such things with an inevitability that is uncharacteristic, but when the alternative of being forcibly injected with the prescribed drugs was presented to me, I caved in. Since that time, I have seen what happens when a patient on the ward “kicks off”—the emergency alarm summons every nurse and healthcare worker to the room of the guilty party, and all hell breaks loose. Kicking and screaming get you nowhere, and being down to under 43 kilos and by nature a coward, I was not prepared to be held down by a team of sturdy medics. Still, I am dopey from last night’s drug, which is a relief after months of no sleep and ongoing agitation culminating in hospitalization. Such is my present life. I wrestle with the tangled bed sheets and turn over for a brief nap. Seven-thirty. Time for a shower, but I have no clean towels. You have to ask for everything in this place; I never went to boarding school (a secret adolescent dream of mine) but the ward is feeling increasingly like one. Well, a mixture of hospital, boarding school, and prison, the latter not helped at all by COVID-19 rules preventing anyone except staff coming and going from the ward, and the removal of iPhones for any “misbehavior.” I pull back the blue curtains and peer out. The windows here are opaque and the only view of the local football pitch is through the black wire mesh that is thick enough to be escape-proof. Lockdown also means no visitors and no walks in the grounds, although those who are able to make use of an adjacent gym and a very small garden when staffs are available to supervise. As usual, the shower room is cold, and there are no hooks to hang cloths or towels upon. This and the trickle of water make a long, leisurely soak out of the question. I had, however, been shocked into some kind of realization of my unappealing appearance by a nurse waving her finger at me and declaring “Wash your hair love, you look awful!” Even mad people can have some sense of pride it seems. Mary, for instance, dresses up in her red frock and gloves and carries a handbag full of old letters and paper clippings.

After a speedy shower, its breakfast time. Someone in their wisdom has decided to drop porridge from the menu but fortunately, one of the staff has her own stash of instant oats and, looking at my weedy body, takes pity on me and heats some up in the microwave. The recent prescription of the antipsychotic Olanzapine has given me a voracious appetite—just as well as I was close to starvation on arrival. The drugs have also made this hospital stay a starker reality as the nurses no longer seem to be acting out my bizarre fantasies but appear as real flesh and blood with thoughts and feelings. The downside of this realization is that I am now highly embarrassed by my apparent notoriety—“so I gather you are a lecturer in psychology” one student nurse declared yesterday. I beg her to promise not to spread this fact around. The psychiatrist, or “the big man” as I prefer to call him, declared some knowledge of my papers, a fact I partly denied because—as I explained—I had ceased to be that person. That is what psychosis does—it plays with your identity such that you believe yourself to be transformed into an agent capable of changing fate—for instance on day three of my admission, catching COVID-19 and dying within a week seemed not so much a worry as a dead certainty. I was appalled that some of the nurses pulled down their masks and others hugged patients; but then being masked up all day must be no joke. As a COVID-19 assessment center, we are tested every two days on arrival, even so it took me a while to realize that I was unlikely to catch anything just because I had anticipated it happening.

Although feeling rather better now, I am anxious today because I am waiting to see the big man about my medication and possible discharge. He is very rational, straight to the point, and business like. Some of the patients think he is cool because he has got long hair and an earring, but personally, I am wary of his power over us. The second time I saw him he increased my medication, something I accepted with a heavy heart. At least he sees me as human, whereas I was asked if I was evil by Darren a fellow inmate who is alternately a messenger of God or God himself. Darren reads the bible most of the day, but his hooded eyes have been on me since I arrived. I have taken to wearing a facemask when I go past him as I am somewhat afraid that I might be who he says I am. Fortunately, he is turned nicer since his friend Alice arrived. Alice is wafer-thin, given to going into trances, dresses in a leopard skin onesie, and was once barred by staff from entering the communal area in “scanty” clothing. She looks 18 but on questioning is older. Alice has declared her love for me—I have returned this compliment by sketching her and gifting her my new pair of pajamas that are too small for me.

The dining area is quiet except for a couple of lone patients at different tables. Stephanie asks if I can join her for company. She has been diagnosed with autism and talks sparely but gets anxious when she is alone. She is now a voluntary patient which means she could leave if she chose to but prefers to be here than on her own in a flat. At some point, she is hoping to get a place in shared accommodation, but mental health is the province of the young, with placements for the middle-aged harder to come by. My young room neighbor Nina, for instance, left yesterday when a place came up in a private facility. I was a bit scared of her at first as she ransacked her room on day two of my sojourn and had the entire nursing crew in her bedroom. In fact, Nina turned out to be a pussy cat, whose main defect was swearing at the dinner staff when she was offered food that in theory might contain nuts. Her allergy to nuts has made her famous here—I heard the story about her anaphylactic episode several times from Kalia, a Middle Eastern girl known for her high-pitched laughter and singing, which trickles like a water fall into my brain as she marches up and down the corridor half the day. Every so often Kalia lets it be known that she hates so and so because they are not her friends, they are “prize bitches.” Swearing here is such a regular occurrence that it is largely ignored, except when accompanied by violence.

After breakfast of two bowls of cereal, it is time for morning medication. Nurse Michael summons me as I attempt to sneak past the clinic room. I was a notable “refusenik” at first—but after three days of pestering and warnings, I finally caved in, and the nurses are delighted at my acquiescence. The clinic routine is familiar now. First off are “obs”—blood pressure, pulse, and weight—then its pills swallowed down by a plastic cupful of water. Strangely, I have come to accept this and can now chat casually with the staff rather than fret about it all. The morning pill can make me sleepy but docile is the preferred mode here.

Ten AM and it is time for a game of Scrabble with Timothy. He declares himself to be the spirit of love and light and thinks that the doctors are experimenting on us all. I’m not sure about the former statement but I partly agree about the experimentation thing. He does not trust the big man, but he likes the female doctor Elina because she has a “good aura.” Some days Timothy’s “meds” make him fall asleep while playing but today he seems keen to start. It’s good to keep him occupied because he is prone to destructive impulses which for two days rendered the communal kitchen out of bounds after several mugs were smashed and sachets of tea and sugar spilled on the floor. He also likes to hide the TV remote which causes irritation among other patients. But he is usually polite to me, and he has a formidable brain for math which helps with the scoring.

At 12.30 it is lunchtime, and we form an orderly queue at the counter. Les, the catering man, shouts out for Cherrie to sing him a song. Cherrie is the ward’s glamor queen who was something of a guardian angel to me on arrival. Despite my antisocial demeanor, Cherrie offered to include me in various pre-Christmas pampering activities, such as facials, hair styling, and nail painting. Sadly, I was not up to any of these pursuits but was touched by her kindness. Since then, I have won a few friends by sharing around my herbal teas and cookies. Cherrie’s story is a sad one; three years ago she was assaulted by a former boyfriend resulting in several broken teeth and she has never forgotten it. Usually, she is singing or laughing but any reminder of this incident puts her into a state of high distress, which can result in violent outbursts and further medication.

On the acute ward, there are around 35 patients with problems of one kind or another. A new arrival is a stunningly attractive East European girl diagnosed with depression who has a ravenous appetite and pours sachets of sugar into her mouth. At least two of the male patients are in love with her, one of whom follows her around and copies whatever she does, but it doesn’t seem to bother her in the least. Some of us suspect that she has seen a lot worse as at the sound of a distant police siren she shouted out “No, no, I’m not a prostitute!” in her broken English. Another ghostlike figure on the female side of the ward is Viola, mother of a young child who works in emergency services. Viola first appeared in the corridor in a ward gown like a phantom, having remained in her room for several days. She rarely makes eye contact and appears to live on coffee and toast—I suspect she cannot bear the noise in the dining area as she wears earplugs all the time. When asked why she was in there she said simply that she had “seen too much.” A proficient artist, she now spends much of her time in the art room—the constant presence of one or two nurses suggesting to me that she might be considered a danger to herself.

Finally, I should mention Melissa, a middle-aged woman with unruly hair who was picked up by the police after writing off her car during an episode of mania. Melissa arrived like a storm, surrounded by nurses and proudly showing off the bruises on her arms—the result of a tussle with some large policemen. Besides one weird conversation in which she declared herself to be the Queen and shared her ambitions to become a nun, my conversations with Melissa are remarkably upbeat and pedestrian. Mood-wise, we are chalk and cheese, I (as I am later informed) have “the face of a lemon,” whereas Melissa seizes every opportunity to spread her gaiety and mischievousness around the ward. Melissa’s friendship is for me a turning point; she refuses to let me slouch in a corner and soon I am back in convent school mode; defying the nurses or tricking them into giving us free vapes to distribute to those who use them. It is silly, prankster behavior, but it stirs something in me and before I know it, I am asking the big man to consider discharging me.

Two days later I am packing for my departure. Flaunting the rules of social distancing, I embrace two of my female companions and high-five the men I have befriended. Two patients are leaving today and another tomorrow, so we celebrate with a final cup of coffee together. There is an atmosphere of anticipation and trepidation—what will life be like beyond the locked doors, will I be able to survive without my institutional routine? The final departure is rapid, I am ushered out and away I go into the fading light of a winters’ day.

## Discussion

Using emotional recall and narrative interpretation, I have shared with readers some recollections of my time in a psychiatric facility. My writing up of this account began within days of leaving the hospital, hence there is no prologue to the institutional narrative, except the paper itself. My subjective construct of acute psychosis, like Campbell’s (2000), was at that time deeply moral one, wherein “battling with one’s demons” was no longer metaphorical but a stark and vivid reality. Already in a hypersensitive state, many of my fears concerning life on a psychiatric ward turned out to be true; they are noisy, busy places where polydrug therapy is virtually mandatory, and the freedoms of the outside world (even during lockdown) become a distant reality. As in other accounts of initial episode psychosis (Griffiths et al., 2019), being admitted to a psychiatric facility for the first time proved to be distressing and disorientating. In the first few days I was terror-stricken about being among the “mad,” but once I came to accept my own place in that institution, I was able to reconnect with humanity and by doing so came to reconnect with myself. On the ward I discovered many things, but what stood out for me was the kindness among peers and the solidarity and generosity which was possibly deepened by our shared experience of pandemic lockdown.

Admission into a psychiatric unit is, for the person concerned, a critical event. Separated and detached from their previous social structure, the patient crosses a physical and psychological threshold (limen), is stripped of aspects of their previous identity, and thrust into a discrete social world, analogous to a human melting pot. This cultural manifestation of liminality is not just the corporeal “liminal space between health and illness” of which Defenbaugh (2008, p. 1402) writes; it is a collective experience that affects all those in the temporary peer group, in this case, residents on an acute psychiatric ward. Personal examples of “communitas” during my time in the hospital included communal eating, playing board games, queuing up for medications, and joining a patient-led karaoke session. That is not to imply that psychiatric units are holiday camps. The title given to cleaning staff of the “hotel management team” had a definite ring of irony, and once I had dismissed such denominators as trickery, they greatly amused me. A very real aspect of my stay on the ward was boredom, and the occupational therapy pursuits designed to break up the day struck me as simplistic and repetitive such that much of the time most patients stayed in their rooms, watched TV, or sat in the corridors passing the time by eating or listening to music. Like Gray (2019), I would advocate more creativity and more therapeutic contact between patients and staff, but also a recognition in the literature that relationships (positive and negative) are primary features of psychiatric units and are significant to patients’ individual and conjoined trajectories.

Again, this is not to glorify life in a psychiatric unit. Aspects of my experience, such as minor bullying, patient squabbles, and acts of rebellion, were reminiscent of the autobiographical book/film “Girl Interrupted” (Kaysen, 1995) and even the classic “One Flew over the Cuckoo’s Nest” (Kesey, 1962). While communitas has been largely used to describe a group’s enjoyment in sharing common experiences such as music or games (E. Turner, 2012), in a previous paper, I wrote of the possibility of a “dark communitas” based on collective grievances which, due its potentially disruptive influence, would pose a threat to the authorities presiding over the established structure (Fixsen & Ridge, 2019). Here, the term “dark” relates to the counter-establishment aspect of the communion, something which I became well acquainted with in the psychiatric care system. Instances in the hospital included exchanges among patients about such things as unhelpful nurses, the indignities of enforced medication, forced removal of computers or mobile phones, and other tales of woe. Yet, despite such grievances, I met several patients who viewed their time on a psychiatric ward as far preferable to life alone and who were waiting to be accepted to some more permanent communal unit.

In societal terms, there are strong reasons for those in psychiatric institutions to feel side-lined. Policies designed to treat the mentally ill in the community have only enhanced the catastrophic image and stigmatization of being detained in a modern psychiatric institution. Lelliott and Quirk (2004) wrote that the most pessimistic view of acute psychiatric ward hospitals is as nontherapeutic “dumping grounds” for service users who cannot be managed through community services (p. 297). Their review suggests a need to rethink the purpose and function of psychiatric wards, and in particular, moving away from the centrality of pharmacological interventions toward an emphasis on improving staff/patient relationships in these institutions. In the final section, I argue for a relational conception of recovery, including as part of a shared rite of passage, and explained the possibilities for “aggregation.”

### Recovery as Relational

Although in the 21st century a purely biological model of recovery from mental illness has been largely discounted, psychosis is sometimes framed as the exception, with ongoing medication and—in more severe cases—hospitalization, widely regarded as essential for safety and recovery (Bethall, 2009). There is growing recognition of the benefits of involving those with mental illness in the reframing of their own recovery narratives (Fixsen & Ridge, 2017; Petros & Solomon, 2021), yet those removed from “normal” society are generally deemed less capable of independent, coherent thinking. Based on his experiences of decades working in psychiatric facilities, Bethall’s (2009) account of the sequence of events befalling patients who enter these wards bears remarkable similarities to my own story (see pp. 10–13). In particular, he comments on the potentially intimidating nature of the clinical review, exacerbated by the substantial power imbalance between patient and psychiatrist and the brevity of their encounters with each other. These kinds of “doctor/patient, healthy/ill, clean/ unclean binaries” (Defenbaugh, 2008, p. 1420) have not been wholly ignored by the psychiatric profession who have responded by placing more emphasis on person-centered care in their training program, such as in “affording people dignity, compassion and respect” (Royal College of Psychiatrists, 2018, p. 5).

The quality of the patient–practitioner relationship, while important to recovery, is just part of the equation. Views on what constitutes recovery for patients may differ from that of professionals who treat them, with the former placing more importance on sociocultural influences impacting on illness and recovery (Cheshire et al., 2021). Both illness and recovery are multi-relational, and those in instutions are especially subject to the influence of disparate others. In one qualitative study of service users in a psychiatric hospital, relationships formed the core of service users’ experiences. When participants talked about their experiences of being in hospital they did so in the context of the people they had encountered since their admission. In general, participants felt more listened to by other service users than staff (Gilburt et al., 2008). Yet, perhaps because of the eclectic nature of ward relationships, the ways in which psychiatric patients might form meaningful associations within psychiatric units remains largely absent from the recovery/survival literature. Recovery (whether in or outside of institutional settings) is still largely viewed as an individualized process in which social life and relationships play a secondary role (Price-Robertson et al., 2017). Gray’s account of peer support in a mental health hospital from a service user’s perspective, in which he frames this as a shared experience and journey is one exception (Gray, 2019). “The main reason I started to feel better was as a result of the friendships on the ward” he says (p. 282). Writing of her relationships in an asylum, Taylor (2014a) speaks of how the obligations of friendship “trumped” madness, and “this in itself could be a form of healing.”

A further way of considering recovery is more symbolic, but is equally a relational process. Returning to Turner’s conceptualization of a rite of passage, the stage of “aggregation” can be interpreted in several ways. In the context of this study aggregation could mean the physical act of being discharged from the hospital and all that this implies; the reconfiguration of selfhood as different (better or worse) from how it previously existed; or as I prefer to interpret it, as the collecting, amassing, amalgamating of diverse but overlapping emotions and experiences that, once separate, have become part of partakers’ perceptions of the world. My time in hospital has certainly altered my views of collective human experience, in many ways for the better; if nothing else, it has confirmed for me that recovery is never a solely medicinal or individual endeavor but something that can even happen despite these things. I hope that in this article I have given some impression of the complex nature of the acute psychiatric hospital, not only as a place of enforcement but also one of the relational possibilities, some of which (in particular the impact of informal peer to peer interactions) have been neglected in “recovery” studies of mental illness and almost entirely overlooked by conventional psychiatry.

Finally, I would like to add some thoughts concerning the isolating effects of serious mental health problems, especially in times of extremis, such as during the current pandemic. As with all critical occurrences, acute psychosis creates an ultra-consciousness, a separation from the people and objects that inhibit what is widely regarded as normal life. Under COVID-19 lockdown, normality itself shifted, and while in some areas this was characterized by demonstrations of solidarity (such as in “Save our NHS”), for many individuals it has proved to be a truly isolating and disorientating experience (Kozloff et al., 2020). For the future, it seems important that services, even when stretched, safeguard against psychological and social isolation by creating more spaces for struggling people to interact without fear or prejudice.
